# Increased prevalence of *EPAS1* variant in cattle with high-altitude pulmonary hypertension

**DOI:** 10.1038/ncomms7863

**Published:** 2015-04-15

**Authors:** John H. Newman, Timothy N. Holt, Joy D. Cogan, Bethany Womack, John A. Phillips, Chun Li, Zachary Kendall, Kurt R. Stenmark, Milton G. Thomas, R. Dale Brown, Suzette R. Riddle, James D. West, Rizwan Hamid

**Affiliations:** 1 Department of Medicine, Division of Allergy, Pulmonary and Critical Care, Vanderbilt University School of Medicine, Nashville, Tennessee 37232, USA; 2 Department of Veterinary Sciences, College of Agricultural Science, Colorado State University, Ft Collins, Colorado 80523, USA; 3 Department of Pediatrics, Division of Medical Genetics and Genomic Medicine, Vanderbilt University School of Medicine, Nashville, Tennessee 37232, USA; 4 Department of Epidemiology and Biostatistics, Case Western Reserve University, Cleveland, Ohio 44106, USA; 5 Cardiovascular Pulmonary Research Laboratory, Department of Medicine and Pediatrics, University of Colorado, Denver Colorado 80045, USA; 6 Department of Animal Science, Colorado State University, Ft Collins, Colorado 80523, USA

## Abstract

High-altitude pulmonary hypertension (HAPH) has heritable features and is a major cause of death in cattle in the Rocky Mountains, USA. Although multiple genes are likely involved in the genesis of HAPH, to date no major gene variant has been identified. Using whole-exome sequencing, we report the high association of an *EPAS1* (HIF2α) double variant in the oxygen degradation domain of *EPAS1* in Angus cattle with HAPH, mean pulmonary artery pressure >50 mm Hg in two independent herds. Expression analysis shows upregulation of 26 of 27 HIF2α target genes in *EPAS1* carriers with HAPH. Of interest, this variant appears to be prevalent in lowland cattle, in which 41% of a herd of 32 are carriers, but the variant may only have a phenotype when the animal is hypoxemic at altitude. The *EPAS1* variant will be a tool to determine the cells and signalling pathways leading to HAPH.

## Abstract (web summary)

Pulmonary hypertension and congestive right heart failure afflict some cattle living at high altitude in an autosomal dominant pattern, yet no responsible genes have been identified. Here Newman *et al.* use whole-exome sequencing to identify variants in the hypoxia inducible factor gene, EPAS1.

## Graphical abstract

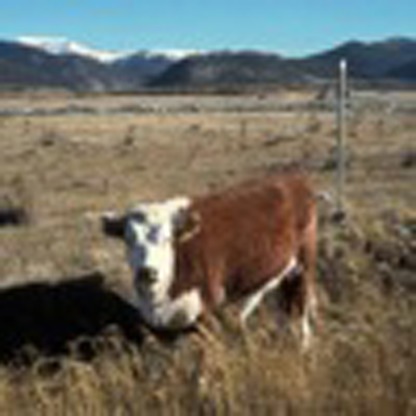

High-altitude pulmonary hypertension (HAPH) in cattle leads to Brisket Disease, which is congestive right heart failure with oedema of the chest muscles and gravity-dependent tissues^1^,^2^,^3^,^4^,^5^,^6^,^7^,^8^,^9^,^10^. Bovine HAPH is heritable in an autosomal dominant mode with incomplete penetrance^7^,^8^,^9^,^10^. The responsible genes are unknown. Brisket disease was first recognized in high altitude ranching in the early 1900s^1^,^9^. Evidence that HAPH in cattle is an autosomal dominant trait was suggested by breeding experiments by Grover and Reeves^3^,^4^,^7^,^8^. A few studies have investigated the genetic basis of HAPH but no genes with a high likely impact have been identified^6^,^11^. Epidemiological studies in high-altitude cattle have found influences of age, gender, birth weight and growth rate in addition to heritability. Early death in high-altitude calves is also associated with undiagnosed pulmonary hypertension^10^,^12^.

Bos taurus cattle have one of the highest acute and chronic hypoxic pulmonary arterial pressure (PAP) responses of all species^2^,^3^,^4^. Cattle have a basal sea level mean PAP of approximately 27–29 mm Hg, versus only 14 mm Hg in human. There is no strict definition of pulmonary hypertension in cattle, but at age greater than 12 months and residence at or above 7,000 feet altitude, a mean PAP of 31–41 mm Hg is considered normal, and above 49 mm Hg is considered at high risk for brisket disease^11^,^13^,^14^. PAPs between 42 and 48 mm Hg are in an uncertain zone. PAP rises with duration at high altitude, the degree of altitude and age of the animal. Some animals develop HAPH at altitudes near 5,000 feet. HAPH affects 5–10% of most and up to 50% of some herds moved from low altitude to >7,000 feet altitude ranches^5^,^7^,^11^,^13^,^14^. Affected cattle may die of right heart failure if not identified and moved to lower altitude. Over 2 million head of cattle reside at high altitude in the United States of America and the annual losses to high-altitude ranches are considered substantial, estimated at multiple millions of dollars a year^5^,^13^,^14^. There is no known measurement at low altitude to predict which cattle will develop HAPH upon ascent. The high rate of development of HAPH in cattle shipped to high altitude suggested us that the variant was common in lowland herds. Human HAPH is a worldwide problem, with over 140 million high-altitude dwellers at risk^15^,^16^. We describe a newly identified double variant in the hypoxia inducible factor (HIF2α) gene, *Endothelial PAS domain-containing protein 1* (*EPAS1)*, that is highly associated with HAPH and probably represents a gain of function mutation that is prevalent in low-altitude Angus herds and only expresses disease in the hypoxic state at high altitude.

## Results

### Phenotype data and initial whole-exome sequencing (WES)

**Figure 1:**
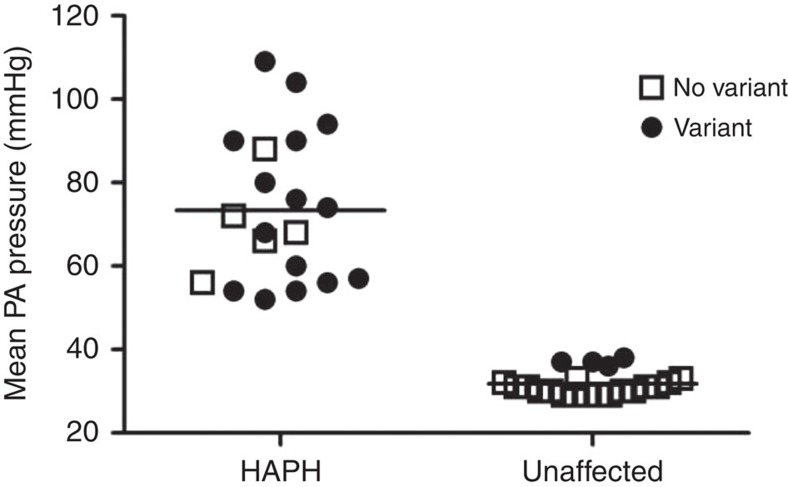
Mean pulmonary arterial pressure in 41 Angus cattle dwelling at high altitude. Of the 20 HAPH (high-altitude pulmonary hypertension PAP>50 mm Hg), 15 carried the EPAS1 variant and all of the cattle with the highest PAP carried the variant. Four of the 21 unaffected cattle carried the variant, and these 4 had the highest pressure, albeit normal PAP <38 mm Hg of the group.

We identified 41 (30 bulls and 11 cows) Angus cattle residing at high altitude from three ranches at altitudes of 4,850, 7,200 and 8,590 feet. All cattle underwent right heart catheterization by an experienced veterinarian (T.N.H.) at rest to measure PAP and draw blood for DNA testing. Twenty cattle had HAPH with PAP >50 mm Hg and twenty-one cattle had PAP <39 mm Hg. Our strategy as follows: we initially used comparative WES analysis of DNA from five cattle with extreme HAPH versus five unaffected controls to seek any important variant. To ensure the selected cattle were as genetically diverse as possible, we genotyped ten microsatellite markers on all DNA samples and calculated pairwise ‘distance’ score, calculated as the number of alleles that differed between samples, using SimpleSeq2 (ref. 17). The five cattle with the highest diversity in the high PAP group had mean PAP of 94.2±5.3 and the five cattle with the highest diversity in the low PAP group (unaffected) had mean PAP of 30.8±0.8 (Fig. 1). We generated an average of 10 Gb of sequence per animal as paired-end 100 bp reads by WES with 80-fold coverage. About 97% of the target regions had sufficient coverage to pass our thresholds for variant calling. Owing to small initial sample size, we focused only on non-synonymous variants, and short coding insertions or deletions.

### Filtering sequence

**Figure 2:**
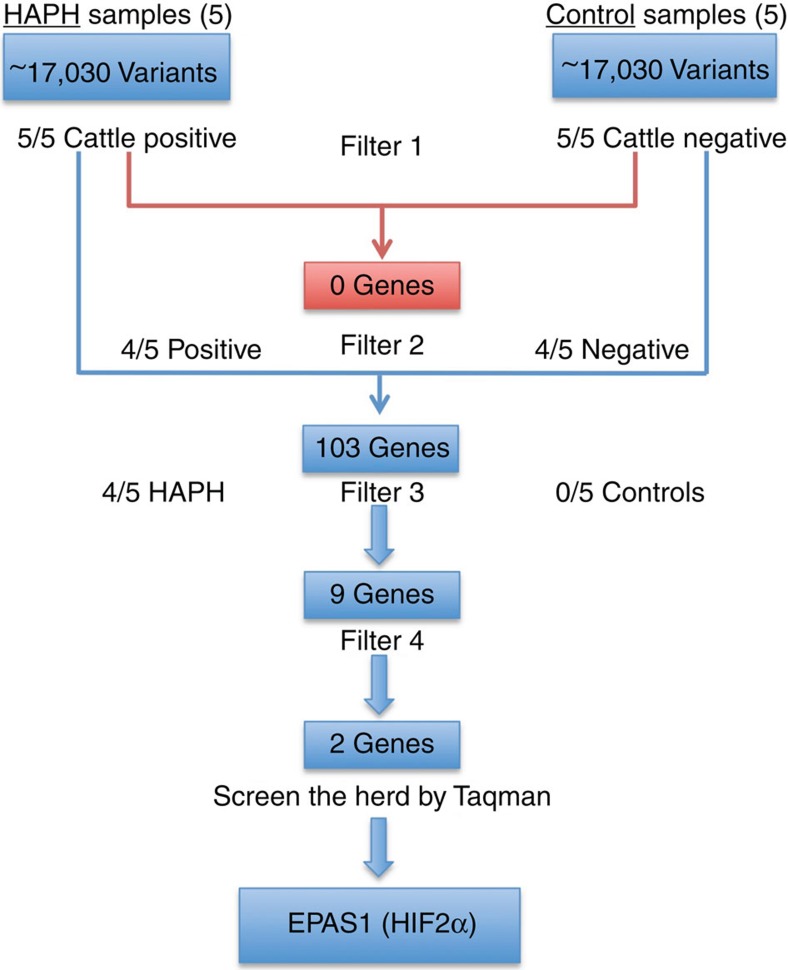
Sequence of filtering and analysis of WES data using an autosomal dominant model. First filter was variant found in all five HAPH and in none unaffected. Second filter was the presence in 4/5 HAPH and 1/5 affected, yielding 103 genes. Filter 3 was 4/5 HAPH and 0/5 unaffected, yielding 9 genes. Sanger sequencing reduced the number to two candidates, *EPAS1* and *PDPR*. Taqman assay revealed no association with *PDPR*, but highly significant association of HAPH with *EPAS1* variant.

**Figure 3:**
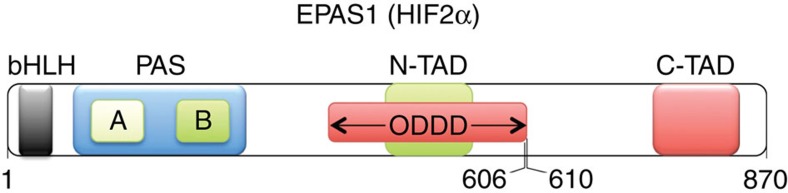
Graphic depiction of the known *EPAS1* domains. bHLH, basic Helix–Loop–Helix; PAS, Per-Arnt-Sim domain; ODDD, oxygen-dependent degradation domain; N-TAD, N-terminal transactivation domain; C-TAD, C-terminal transactivation domain. The two variants are in the ODDD domain of the protein.

We used an autosomal dominant model for screening (Fig. 2). We used a simple algorithm that showed at least four of the five affected samples carry the same variant and four of the five unaffected samples be homozygous negative and for a candidate variant. This generated a list of 103 genes (Supplementary Table 1) with a sequence variation, of which 9 genes (Table 1) were found to be homozygous negative in all unaffected and heterozygous positive for a variant in four of the five of affected. Sanger sequencing of ten additional samples, five affected and five unaffected, narrowed the list to two genes, *EPAS1* and pyruvate dehydrogenase phosphatase regulatory subunit (*PDPR*), in which none of the ten unaffected and eight of the ten affected samples had the variant. *EPAS1* contained two variants in close proximity to each other, c.1816G-A and c.1828G-A, both in exon 12. Each sample had either both variants or none, indicating that both were in haplotype. The *EPAS1* variants predicted two non-synonymous substitutions, p.A606T and p.G610S, in the oxygen-dependent degradation domain of the HIF2α protein (Fig. 3).

We then designed Taqman assays for the 2 *EPAS1* variants and the 1 *PDPR* variant, and genotyped 10 additional HAPH cattle and 11 additional unaffected animals to fully evaluate the 41 variants. The 4 unaffected cattle that carried the variant had the highest PAPs among all 21 unaffected (36–38 mm Hg), at the upper limit of normal. Taking all 41 cattle, 15 of the 20 samples with PAP >50 mm Hg were positive for the two *EPAS1* variants and 4 of 21 with PAP <39 mm Hg had both variants, with a *χ*^2^ *P*-value of 3.3 × 10^−4^.

Armitage trend test for this herd was 1.8 × 10^−3^. Wilcoxon’s rank sum tests on PAP between EPAS1 variant carriers and non-carriers were also significant, *P*=4.6 × 10^−5^. *PDPR* variant did not segregate with high PAP; 10 of the 20 affected samples and 5 of the 21 unaffected had the *PDPR* variant (*P*<0.08). With a likelihood of development of HAPH at 50%, the positive predictive value of the EPAS1 variants would be 79.7% and the negative predictive value would be 76.4%. The relative risk for HAPH between a carrier and a non-carrier is estimated to be 3.47 (1.55–7.77), and the odds ratio is estimated as 12.75 (2.9–66.4). Trend test *P*-value was 0.002. We found no variants that segregated with HAPH in a number of known PH genes, including *BMPR2, ACVRL1, Cav1, VHL* and endoglin. There were no other non-synonymous variants in *EPAS1* in the cattle.

### Replication in second herd and lowland cattle

We tested and replicated the variant data in a second herd, residing at 7,100 feet in Wyoming. These were young cattle, studied about 6 months of age, but they had been born and raised at this altitude^18^. Eleven of the fifteen cattle with PAP ≥45 mm Hg but only four of sixteen with PAP ≤39 mm Hg carried the variant, *χ*^2^ *P*-value<0.007 (Supplementary Table 2). A joint analysis of the original herd (20 affected and 21 unaffected) and the new replication herd has a *P*-value 7.7 × 10^−6^ by *χ*^2^ test. Despite a small number of cattle, this exome-wide survey found a variant of very high association with HAPH, which was replicated in a second independent herd. Although the original *P*-value was not as strong as in typical genome-wide association studies due to the small sample size, the replication finding was statistically highly significant, and the odds ratio suggests a strong effect of the variant in HPAH.

Because of the high rate of development of HAPH in lowland cattle moving to high altitude, we predicted that the variants would be present at a reasonably high frequency in lowland cattle. To test this hypothesis, we obtained DNA samples from 32 Angus cattle residing in Alabama, USA, to screen for the two *EPAS1* variants. In all, 13 of the 32 samples had both variants (41%), with every positive sample carrying both variants.

### Conservation among species and stability

Since the two variants introduce non-synonymous changes in the HIF2α protein, we investigated existing databases to see if the corresponding residues are conserved among species. These two amino acids are well-conserved across species. Interestingly, yak, which has a nearly identical HIF2α protein sequence to cattle and has adapted to high altitude, is not reported to have either of these changes (http://www.mevolab.net/yak). Sheep are known to have one of the two single-nucleotide polymorphisms found in cattle, at codon 606, but not the second variant. Sheep are much less susceptible to HAPH than cattle. Given that all affected bovine have both the variants, it is possible that both changes need to be present together to alter function of the protein. Another possibility is that the HIF pathway is configured in sheep to tolerate one variant allele.

Mutations in the same oxygen-dependent domain in the human *EPAS1* gene show increased stability and decreased degradation associated with increased downstream transcriptional activity, consistent with a gain of function^19^. We used computational tools to predict the function of the variants residues in cattle. The analysis showed that each variant was predicted to significantly increase the protein stability of HIF2α. Two stabilizing variants together in *cis* are predicted to result in an even more stable protein. We note that the two residues were in perfect linkage disequilibrium in our cattle. It is still possible that the variants are in linkage disequilibrium with another variant of effect on the hypoxic response.

Since these variants were predicted to increase HIF2α stability, we hypothesized that this would result in upregulation of downstream HIF2α target genes. We thus looked at differential expression between peripheral blood mononuclear cells from cattle with HPAH and HIF2α variants and unaffected cattle without HIF2α variants by analysing our previously published gene expression array data^6^. We found that in cattle containing the double HIF2α variant, transcription of HIF target genes was significantly overrepresented (*P*<0.0001 by *χ*^2^chi-square test), with 26 of 27 HIF target genes significantly upregulated compared with unaffected cattle (Supplementary Table 3).

## Discussion

Using WES and an autosomal dominant model in 20 HAPH and 21 unaffected Angus cattle living at high altitude, we found two variants, in *cis*, in exon 12 of the *EPAS1* gene that encodes a hypoxia inducible factor, HIF2α. The variants were present in 75% of the 20 cattle with mean PAP >50 mm Hg, in all 5 cattle with extreme HAPH (PAP >94 mm Hg), and in 19% of 21 unaffected cattle with PAP <39 mm Hg. We replicated the finding in a second, unrelated herd from Wyoming, in which 11 of 15 cattle with HAPH and only 4 of 16 unaffected were carriers^18^. We also found higher mRNA expression in variant carriers with HAPH than in unaffected 26 of 27 genes, known to be targets of HIF2α^6^. This double variant haplotype in *EPAS1* is likely a gain-of-function mutation that requires a gene–environment interaction for expression.

Human EPAS1 and HIF1α are basic helix–loop–helix transcription factors that contain a Per Arnt Sim (PAS) domain and share 48% sequence homology^20^,^21^. During sufficient oxygen availability, the resulting HIF proteins are constantly degraded, but are released for function during cell or tissue hypoxia. Under hypoxic conditions, HIFs are protected by inhibition of oxygen-dependent hydroxylation of specific residues in the oxygen-dependent degradation domain. This prevents interaction with the von Hippel–Lindau ubiquitin ligase complex and proteasomal destruction. HIF2α is found in all human tissues including lung and lung vasculature. The downstream effects of HIF2α include regulation of angiogenic factors including VEGF and TGF-α, and cell permeability and stimulation of erythropoietic and glycolytic proteins^20^,^21^,^22^.

Rare germline *EPAS1* gain-of-function variants can cause familial erythrocytosis and/or pulmonary hypertension^23^,^24^. A von Hippel–Lindau mutation that reduces destruction of HIF2α is found in Chuvash populations and is associated with modest pulmonary hypertension and erythrocytosis^25^. Transgenic mice carrying a G536W variant in *EPAS1* developed high right ventricular systolic pressure and medial hypertrophy of pulmonary arteries in addition to erythrocytosis under normoxic conditions^26^. Thus, HIF2α dysregulation is well recognized in association with pulmonary hypertension, frequently in context of erythrocytosis, with or without severe hypoxemia.

In contrast, probable loss-of-function variants in EGLN1 and HIF2α were found to be enriched in residents of the Tibetan Plateau^27^,^28^,^29^. Native Tibetans are recognized to have beneficial adaptation to high altitude, with less polycythemia, less haemoglobin desaturation, preserved ventilatory responses and normal birth weights. The inference from these studies is that loss of function of HIF2α driven either directly or indirectly by downregulation of modifying proteins may be beneficial in states of chronic hypoxia where chronic HIF protein stabilization may be detrimental.

In summary, we found that two *cis* variants in *EPAS1* (HIF2α) are highly associated with HAPH in Bos taurus Angus cattle residing at high altitude in the Rocky Mountains, USA. Given what is known about HIF2α function, our data suggest that *EPAS1* is likely a major gene for HPAH in cattle. Other genes are undoubtedly involved in bovine HAPH. Additional studies are needed to clarify its biological role in HAPH in cattle and any role of the variant in human HAPH.

## Methods

### Collection of samples and measurement of PAP

We (T.N.H.) obtained blood from Black Angus cattle of both genders from four sites, aged 12–18 months, residing at 5,200–7,850 feet, by jugular vein puncture on the same day of right heart catheterization, done to screen for HAPH, and blood from an Alabama herd of cattle residing near sea level. The project was approved by the Vanderbilt Medical Center, Institutional Animal Care Use Committee.

### DNA isolation and microsatellite-based genotyping

DNA was isolated from whole blood using QIAmp DNA mini-kit as directed by the manufacturer's instructions (Qiagen) and subsequently quantified using a spectrophotometer. Samples were genotyped using the StockMarks for Cattle Bovine Genotyping kit (Cat. Number 4307480; Life Technologies).

### WES and data preprocessing

We used Agilent SureSelect XT Bovine All Exon kit (Cat. Number 5190–5448; Agilent) to capture the cow exome. The captured DNA library was then sequenced on an Illumina HiSeq2000 instrument. The sequences were aligned to the cow genome bosTau6 (UMD3.1) using Burrows-Wheeler Aligner (http://bio-bwa.sourceforge.net). Duplicates were marked using Picard, and the sequences were locally realigned and base quality score was recalibrated using the Genome Analysis Tool Kit (GATK; http://www.broadinstitute.org/gatk/). Joint genotype calling was performed using GATK, followed by ‘hard filtering’ as recommended on the GATK website. In addition, variations within ten bases from an insertion/deletion site were removed, and genotypes with GQ<30 were removed. The variant calls were annotated using ANNOVAR (http://www.openbioinformatics.org/annovar/).

### Sanger sequencing and Taqman analysis

All DNA sequencing was performed using BigDye (Life Technologies) according to the manufacturer’s instructions. Taqman assays were obtained from Life Technologies. The assays targeted the variations found on WES and were designed so that the wild-type allele utilized the VIC probe and the minor allele utilized the FAM probe. Analysis was done on 5 ng of genomic DNA using ABI PRISM 7500 instrument (Life Technologies) per the manufacturer’s instructions.

### Protein stability analysis

The two EPAS1 variants A606T and G610S were individually submitted to the MuPro website (http://www.ics.uci.edu/~baldig/mutation.html) in conjunction with the Bovine EPAS1 protein sequence (NP_777150) to determine their impact on protein stability. To determine the effect of each amino-acid substitution on protein function, they were submitted to the SIFT (http://sift.jcvi.org/www/SIFT_seq_submit2.html) and Polyphen-2 (http://genetics.bwh.harvard.edu/pph2/) for analysis.

### Gene expression array analysis

Of 171 genes found to be upregulated by hypoxia in the Broad Institute set GSEA Elvidge_Hypoxia_Up^30^, 68 have expression values above 7 log base 2 Affymetrix units in either affected or unaffected data sets (thus, within reliable signal values). Of these 68, 26 (38%) are upregulated in the affected group compared with the unaffected (*P*<0.05 by uncorrected *t*-test, fc>1.25; Supplementary Table 2), whereas only one is downregulated (1.5%). Based on the total numbers of up- or downregulated genes expressed above 7 log base 2 Affymetrix units in the overall data set using the same criteria, our expectation values are 7% upregulated and 8% downregulated (or 5 genes upregulated and 5 genes downregulated) based on random variation. Chi squared is thus calculated as 96.4 with 2 degrees of freedom, for a two-tailed *P* value of less than 0.0001, Supplementary Table 2.

### Statistical analysis

Statistical analysis was done using Prism for Mac.

## Additional information

**How to cite this article:** Newman, J. H. *et al.* Increased prevalence of *EPAS1* variant in cattle with high-altitude pulmonary hypertension. *Nat. Commun.* 6:6863 doi: 10.1038/ncomms7863 (2015).

## Supplementary Material

Supplementary Information Supplementary Tables 1-3 and Supplementary Methods

## Figures and Tables

**Table 1:** Nine genes found in 4/5 HAPH and 0/5 unaffected cattle at altitude.

**Gene**	**Symbol**	**Function**	**Cow chromosome**	**Ref Seq mRNA**	**Non-synonymous variation**	**Location (Btau_4.6.1)**
*Budding uninhibited benzimidazoles 1*	*BUB1*	Mitotic spindle checkpoint kinase	11	NM_001102011	Exon10:c.G1165A:p.V389M	1571369
*Complement component*	*C4A*	Complement factor 4	23	NM_001166485	Exon11:c.A1262G:p.Q421R	27193512
*Feline leukemia virus*	*FLVCR2*	Calcium transporter	10	NM_001192143	Exon9:c.C1239G:p.D413E	87200257
*Leucine-rich repeat-containing protein 17*	*LRRC17*	A negative regulator of receptor activator of NF-κB	4	NM_001078150	Exon2:c.A95T:p.H32L	44501073
*Pyruvate dehydrogenase phosphatase*	*PDPR*	Pyruvate metabolic electron transport	18	NM_174781	Exon3:c.C327G:p.H109Q	1939296
*Regulator of G-protein signalling 18*	*RGS18*	Regulator of G-protein signalling	16	NM_001192971	Exon4:c.C397T:p.R133C	13749384
*Zinc finger MYM-type protein 6*	*ZMYM6*	Regulation morphology and cytoskeleton	3	NM_001206292	Exon16:c.G2834C:p.C945S	111275016
*Endothelial PAS domain-protein 1 (hypoxia-inducible factor 2-α)*	*EPAS1*	Transcription factor in the induction of genes regulated by oxygen	11	NM_174725	Exon12:c.G1816A:p.A606T. Exon12:c.G1828A:p.G610S	28662654
*Rho GTPase activating protein 20*	*ARHGAP20*	GTPase activator for the Rho-type GTPases	15	NM_001206733	Exon15:c.C3262T:p.P1088S	20909547

NF-κB, nuclear factor-κB.
