# Supplementary material for: Increased prevalence of EPAS1 variant in cattle with high-altitude pulmonary hypertension
Source: Nat Commun. 2015 Apr 15;6:6863. doi: 10.1038/ncomms7863 (PMC4399003; doi:10.1038/ncomms7863)
Supplement: Supplementary Information — Supplementary Tables 1-3 and Supplementary Methods [file ncomms7863-s1.pdf]

Supplementary Table 1 : 103 gene variants found in 4/5 HAPH cattle and 1/5 Unaffected

**Bovine Chromosome**	**Bovine Gene**	**Human Protein/Function**
chr25	ABCA3	surfactant transporting gene
chr6	AFAP1	actin filament gene
chr21	AKAP6	anchors a protein kinase to membrane
chr21	ANKRD34C	membrane proteins that promote channels
chr1	AP2M1	acidification protein
chr10	AQPEP	metalloprotein embryonal maternal interface
chr15	ARHGAP20	rho gtpase activating protein
chr13	ARMC3	many basic functions armadillo- cell
chr17	BBS12	membrane trafficking Bardet-Biedle syndrome
chr5	BCDIN3D	miRNA processing
chr23	BTN3A3	histocompatibility gene
chr11	BUB1	mitotic spindle control gene
chr1	C1H21orf33	chicken gene, involved in cancer, not much known
chr23	C4A	complement factor 4 inflammation
chr18	CA5A	carbonic anhydrase, kidney other organs
chr5	CBX6	transcriptional repression gene
chr3	CCBL2	tryptophan metabolism
chr22	CCK	cholecystokinin gene
chr18	CES5A	hydrolysis of xenobiotics
chr25	CLN3	lysosomes neurodegenerative
chr11	COQ4	coenzyme Q2 biosynthesis, neurological
chr13	COX4I2	cytyochrome c oxidase subunit 4
chr23	CRISP1	sperm and egg sticky protein
chr10	DHRS1	basic oxyreductase in multiple pathways
chr19	DHRS13	same as above
chr19	DHX33	DEAD box protein basic cell functions
chr18	DHX34	same as above
chr5	DNM1L	mitochondrial division, Dynamin, neuro
chr9	ECHDC1	toxic metabolite proof reading liver?
chr11	EPAS1	HIF2alpha
chr10	FLVCR2	CNS Ca transporter gene
chr19	FN3K	fructosamine kinase, metabolic
chr5	FOXRED2	endoplasmic reticulum degradation
chr7	GLRA1	CNS post synaptic inhibition
chr3	GON4L	transcription factor not much known
chr2	GORASP2	golgi restacking protein
chr13	GPR158	G protein coupled receptor
chr3	GPSM2	G protein controller deafness

chr23	GSTA3	zinc finger t cells and endothelium
chr17	GSTT1	glutathione conjugation
chr18	HRC	cholesterol binding sarcoplasmic reticulum
chr22	HRH1	histamine H1 receptor
chr29	IFITM3	interferon influenza resistance
chr11	KLF11	diabetes and apoptosis
chr21	KLHL25	ectoderm neural complex gene
chr5	KLRJ1	natural killer cell domain
chr23	LOC525599	butyrophilin family uncharacterized
chr4	LRRC17	Leucine-rich repeat-containing protein 17
chr23	MIC1	Macrophage inhibitory cytokine-1
chr23	MLIP	Muscular-Enriched A-Type Laminin-Interacting
chr1	MRPL47	mitochondrial protein synthesis
chr7	NDST1	Bifunctional heparan sulfate N-deacetylase/N-sulfotransferase 1
chr28	NDST2	same as above
chr22	NEK4	testicular protein of NEK family
chr8	NOL6	nucleolar protein 6
chr10	NRDE2	RNA interference gene
chr2	OSGEPL1	O-sialoglycoprotein endopeptidase-like 1
chr3	OVGP1	OVGP1 oviductal glycoprotein 1
chr7	PCDH12	protocadherin gene family, a subfamily of the cadherin superfamily
chr18	PDPR	PDPR pyruvate dehydrogenase phosphatase
chr16	PHF13	finger protein male fertility
chr18	PNMAL1	paraneoplastic MA antigen family
chr6	PSAPL1	Prosaposin-like 1
chr11	PTGS1	prostaglandin-endoperoxide synthase 1
chr7	RAB3D	RAS oncogene family
chr8	RCL1	RNA terminal phosphate cyclase-like 1
chr7	RFX2	RFX2
chr16	RGS18	regulator of G-protein signaling
chr23	RHAG	erythrocyte-specific
chr29	RIN1	expressed in cancers
chr23	RPP40	ribonuclease subunit for making t RNA
chr3	RPS8	ribosomal subunit protein
chr22	RPUSD3	RNA pseudouridylate synthase domain containing 3
chr28	RTKN2	inflammation, lupus, nf Kappa b
chr10	SEMA6D	Semaphorins
chr6	SHROOM3	Shroom protein family
chr1	SIDT1	transmembrane family
chr8	SLC1A1	neuronal glutamine transporter
chr19	SLC25A11	transports 2-oxoglutarate across mitochondria

chr19	SPAG5	sperm mitotic spindle protein
chr7	SPINK5	serine protease inhibitor
chr3	ST7L	tumor suppressor gene
chr6	TBC1D14	Negative regulator of starvation-induced autophagosome formation
chr5	TBC1D22A	protein homodimerization activity
chr2	TCEB3	transcription elongation factor
chr14	TERF1	telomeric repeat binding factor (NIMA-interacting)
chr11	THUMPD2	methyl transferase poorly characterized
chr22	TMEM42	transmembrane protein nothing known
chr16	TMEM63A	transmembran protein nothing known
chr15	TRIM5	is a retrovirus restriction factor
chr3	TTF2	transcription termination factor, RNA polymerase II.
chr5	TXN2	mitochondrial member of the thioredoxin family,
chr10	UNC13C	poorly characterized membrane diacylglycerol
chr15	USH1C	eye and ear stereocilia
chr16	USH2A	same as above Usher syndrome
chr9	VNN1	hematopoietic cell trafficking
chr28	WDFY4	no information
chr25	WDR24	no information
chr3	ZMYM6	cell morphology and cytoskeletal organization.
chr19	DHX33	dead box protein family cell basic function
chr23	MIC1	already seen above #54
chr7	DNM2;DNM2	Dynamins
chr19	DHX33	dead box proteins as above

Supplementary Table 2

Ear Tag #	*mPAP (mmHg)*	*Zygosity*
2155	64	Heterozyote G/A
2355	54	Heterozygote G/A
2171	53	Heterozygote G/A
2201	53	Heterozygote G/A
2156	48	Wildtype G/G
2200	48	Homozygote A/A
2221	48	Heterozygote G/A
2033	47	Wildtype G/G
2064	47	Heterozygote G/A
2213	47	Heterozygote G/A
2158	45	Homozygote A/A
2204	45	Wildtype G/G
2229	45	Wildtype G/G
2231	45	Heterozygote G/A
2337	45	Homozygous A/A
mPAP below this line ≤ 39		
2052	39	Wildtype G/G
2135	39	Homozygote A/A
2181	39	Heterozygote G/A
2311	39	Wildtype G/G
2409	39	Wildtype G/G
2019	38	Wildtype G/G
2035	38	Wildtype G/G
2039	38	Wildtype G/G
2290	38	Homozygous A/A
2356	38	Wildtype G/G
2111	37	Wildtype G/G
2248	37	Wildtype G/G
2396	37	Homozygote A/A
2349	36	Wildtype G/G
2402	36	Wildtype G/G
2145	35	Wildtype G/G

Supplementary Table 3

Gene Symbol	***Gene Title***	***Fold Change***	***T-Test***
ANG	angiogenin, ribonuclease, RNase A family, 5	1.4	2.4E-02
BNIP3L	BCL2/adenovirus E1B 19kDa interacting protein 3-like	1.4	1.0E-03
CADM1	cell adhesion molecule 1	1.8	3.6E-04
CD59	CD59 molecule, complement regulatory protein	1.9	2.6E-03
CYB5A	CYB5 protein	1.4	1.5E-04
DUSP1	dual specificity phosphatase 1	1.6	1.2E-02
ECE1	endothelin converting enzyme 1	1.4	5.5E-05
FOS	FBJ murine osteosarcoma viral oncogene homolog	1.6	2.0E-02
GADD45B	growth arrest and DNA-damage-inducible, beta	1.5	2.2E-02
GYS1	glycogen synthase 1 (muscle)	1.4	2.0E-04
ISG20	interferon stimulated exonuclease gene 20kDa	1.8	2.3E-02
JUN	jun oncogene	1.7	5.8E-05
KLF6	Kruppel-like factor 6	1.7	3.3E-03
NDRG1	N-myc downstream regulated 1	1.6	1.1E-04
NFIL3	nuclear factor, interleukin 3 regulated	2.5	7.2E-04
PAM	peptidylglycine alpha-amidating monooxygenase	1.7	1.8E-05
PFKFB3	6-phosphofructo-2-kinase/fructose-2,6-biphosphatase 3	1.6	7.2E-03
PLAC8	placenta-specific 8	1.9	2.5E-02
PLAUR	plasminogen activator, urokinase receptor	1.5	1.9E-03
S100A4	S100 calcium binding protein A4	1.3	3.5E-03
SAT1	spermidine/spermine N1-acetyltransferase 1	1.5	8.3E-03
SCARB1	scavenger receptor class B, member 1	1.4	3.6E-04
SERPINE1	serpin peptidase inhibitor, clade E	1.9	6.0E-06
SORL1	sortilin-related receptor, L(DLR class) A repeats-containing	1.6	6.6E-04
TXNIP	thioredoxin interacting protein	1.3	5.2E-03
vldlr	very low density lipoprotein receptor	2.3	3.4E-07
ZMYND8	Zinc finger, MYND-type containing 8	-1.5	7.1E-03

Supplementary Methods

TaqMan Allelic Discrimination Protocol

SNP assays need to be diluted to a 20X working concentration with 1XTE and aliquoted (stock assay concentration is 40X or 80X, depending on the assay ordered).

- 11.25uL gDNA + H2O, with a gDNA concentration of 1-20ng total is needed per rxn
- If possible, two controls are needed
    - Positive control: homozygous or heterozygous for SNP
    - Negative control: NTC, add water instead of gDNA
- If you have many gDNA samples, it is helpful to dilute the gDNA in a 96 well plate. Example: dilute DNA to ~5ng/uL with nuclease free H_2O and use 2uL per rxn (10ng gDNA total).

Setting up reaction:

1X Rxn Mix

12.5uL TaqMan Universal PCR Master Mix (2X), No AmpErase UNG (LifeTech: 4324018)
1.25uL 20X SNP Genotyping Assay
13.75 Total

Make a Reaction Master Mix for all samples; be sure to include NTC and one extra in calculation for pipet, flick tube to mix well, spin down then pipet 13.75uL per well.

In a 96 well plate, pipet:
9.25uL H_2O
2.0uL gDNA (diluted)
13.75uL Rxn Mix
25uL Reaction Volume

Seal plate, lightly vortex, spin down and run on Real Time Machine.

Allelic Discrimination Setup on ABI 7500 Real Time Machine (7500 Software v2.0.4)

Put plate in machine and turn on.
Double click 7500 icon on desktop.
From homepage choose Advanced Setup
- Name experiment starting with the date
- Choose 7500 (96 wells)

- Choose Genotyping for experiment type (click yes on popup)
- Choose TaqMan Reagents
- Choose Standard Run

Experiment Menu

Plate Setup

- Click on SNP Assay1, Edit, Edit SNP Assay
- Name Assay (ex. EPAS1snp1)
- Give Assay ID (ex. Ala606Thr)
- Allele 1 Name or Base(s) (ex. G) Reporter (ex. VIC)
- Allele 2 Name or Base(s) (ex. A) Reporter (ex. FAM)
- Click ok when finished

Assign Sample to Selected Wells

- Enter all sample ID's, click new sample until all samples are entered (label one NTC)
- Assign all samples to plate map on right by clicking a well then checking the box next to the correct sample
- Hi-light all wells that contain a sample, do not hi-light NTC
- Click check box next to SNP Assay, click on the drop down menu under task and choose unknown
- Hi-light the well with NTC, assign the SNP Assay, click the drop down menu under task and choose Negative Control

Run Method

- Change Reaction Volume to 25uL
- Make sure Pre-PCR Read and Amplification are checked (they should be checked by default)

Cycle Conditions:
95ºC 10 min (AmpliTaq Gold Enzyme Activation)
40 Cycles
92ºC 15 sec (Denature)
60ºC 1 min (Anneal/Extend)

Run

- Click on green start button
- Run will finish in ~90 minutes

Run Complete

- Click green Analyze button
- Save file
- Turn machine off

EPAS1 Protocol

GCC>ACC Ala606Thr rs211641317 (no freq info)

GGT>AGT Gly610Ser rs208684340 (no freq info)

tggaagaatgactgagtggaaggagacgcgcaggaaggctggtagcacttgtcagaagaccttccacactttgaagacacatct
gtgttttcctctcttgggcccccaagaccttggcgctgccaggctgtgcaggaggaatgcctcgggcgggagcggggtatgaggggc
taagatgagacagcgtccttgaggcagtgcccactgctgagctgctttgcc**cctgctctgtgtcttctagACTG**ACTTTAACG
AGCTGGATTTGGAGACCCTGGCACCTTACATCCCCATGGACGGAGAGGACTTCCAGCTTAGCCCC
ATCTGCCCTGAGGAGAGCCTCCTGCCGGAGACCCCCCAGTCGGCCCCCCAGCACTGCTTCAGCACC
ATGTCAAACATCTTCCAGCCACTGGCTCCGATGGCCTCTCACAGCACCTTCCTCCTGGACAAGTA
TCAGCAGCAGCTGGAAAGCAAGAAGACGGAGCCTGA**G**CCTTCTTTGAC**G**GTGGGAGCAGGGTGT
CCCTGCTGCAGTGCTGTGGTCAGACCTACACCCCCCTCTCCTCCATGGGGGGCATTTCCAACACCC
AGTGGCCCCCTGACCCACCACTACAGCTGGGGCCCACGAAGTGGCCTGGTGAAGACCGGCACGCA
GAGGCCGTGGGGGCAGCGCCCCTGGGGCTCCCCCCCGCCACACCCCATCTCGCCATGCTCAAGAAG
AGgtcagtgatggagatgctgggctgcttcagctaaggctgtgcagtatgggggaggaggtacagacaggtgcccactaggggca
ggtatggggctccaaaaggcccctggccccatccccagtttccatccgacagaggtgcgatatgccgcttagccttctctcaactctg
agagcttgggctttaggggactcgccctaagatatgtgaggcctcgcgtgggacacattt**tatcagagctggagaccaagaga**

PCR Primers

EPAS1-F **tggaagaatgactgagtggaagg**

EPAS1-R **TCTCTTGGTCTCCAGCTCTGATA**

PCR Product: 986 bp

Seq Primer

EPAS1seq-F **cctgctctgtgtcttctagACTG**

TaqMan SNP1 Assay Ala606Thr:

Forward Primer GCAGCTGGAAAGCAAGAAGAC

Reverse Primer ACTGCAGCAGGGACACC (GGTGTCCCTGCTGCAGT)

Reporter 1 Seq WT_VIC AGCCTGAGCCTTCTT

Reporter 2 Seq VAR_FAM AGCCTGAACCTTCTT

TaqMan SNP2 Assay Gly610Ser:

Forward Primer GCAGCTGGAAAGCAAGAAGAC

Reverse Primer ACTGCAGCAGGGACACC (GGTGTCCCTGCTGCAGT)

Reporter 1 Seq WT_VIC TGCTCCCACCGTCAAA (TTTGACGGTGGGAGCA)

Reporter 2 Seq VAR_FAM TGCTCCCACTGTCAAA (TTTGACAGTGGGAGCA)

PCR using Platinum Taq (for PCR products < 4kb)

1X Master Mix (1µl DNA)

H_2O	18.15µl
10X PCR Buffer, Minus Mg	2.5µl
50mM $MgCl_2$	0.75µl
10mM dNTP	0.5µl
10µM Primer-F	1.0µl
10µM Primer-R	1.0µl
Platinum Taq	0.1µl
	24.0µl

Add 1µl DNA (180 ng)

Final Volume 25µl

Cycling Conditions

94º 30 sec

35 cycles

94º 30 sec

58º 30 sec

72º 1 min

68º 3min

4º ∞

Sequencing Protocol (8-20-2013)

Using 2µl BigDye and 2µl PCR product

Master Mix

3.5 µl H_2O

2.0 µl Big Dye v.3.1

2.5 µl Primer (10 µM)

8.0 µl Total Volume

Add:

ng template used in Seq rx'n based on PCR product size:

0.1 kb PCR: 2 – 3 ng / seq rx'n

0.15kb PCR: 3.5 – 4.5 ng / seq rx'n

0.2kb PCR: 5 – 6 ng / seq rx'n

0.3 kb PCR: 7 – 9 ng / seq rx'n

0.5 kb PCR: 12 – 15 ng / seq rx'n

1 kb PCR: 25 – 30 ng / seq rx'n

2 kb PCR: 50 - 60 ng / seq rx'n

3 kb PCR: 75 – 90 ng / seq rx'n

4 kb PCR: 100 – 120 ng / seq rx'n

5 kb PCR: 125 – 150 ng / seq rx'n

6 kb PCR: 150 – 180 ng / seq rx'n

7 kb PCR: 175 – 210 ng / seq rx'n

8 kb PCR: 200 – 240 ng / seq rx'n

Note: If using less than the recommended amount of template, the amount of Big Dye must be increased.

If reducing the amount of template by 25-30% use 3µl Big Dye (0.5 µl 5X buf)

2.0 µl Exo-sapped PCR product
10.0 µl Total Volume

Using 2µl BigDye and 1.5µl PCR product

Master Mix
3.0 µl H_2O
1.0 µl 5X Seq Buf
2.0 µl Big Dye v.3.1
2.5 µl Primer (10 µM)
8.5 µl Total Volume
Add:
1.5 µl Exo-sapped PCR product
10.0 µl Total Volume

Using 2µl BigDye and 1µl PCR product

Master Mix
3.5 µl H_2O
1.0 µl 5X Seq Buf
2.0 µl Big Dye v.3.1
2.5 µl Primer (10 µM)
9.0 µl Total Volume
Add:
1.0 µl Exo-sapped PCR product
10.0 µl Total Volume

Big Dye Cycling

95°C, 5min

95°C, 30 sec
55°C, 10 sec
60°C, 4 min
30 cycles

Product Cleanup (EtOH Precipitation or Dye Ex)

A. Clean up by Ethanol Precipitation:

1. Remove the 96-well plate from PCR machine and spin down.
2. Add 2.5 µl of 125mM EDTA to each well. Make sure it reaches bottom.
3. Add 30 µl of 100% ethanol to each well.
4. Seal with caps or tape and mix by inverting 4 times.
5. Incubate at room temperature for 15 min.
6. Spin the plate at 4°C at 2885xg for 30 min.

7. Immediately invert the plate and spin up to 180xg, then remove from centrifuge.
8. Add 30 µl of 70% ethanol to each well.
9. Spin at 4°C at 1650xg for 15 min.
10. Invert the plate and spin up to 180xg for 1 min, then remove from centrifuge.
11. Add 10 µl formamide to each well and spin down.
12. Transfer to a 3100 plate and RUN!
    Note: can increase sample uptake on ABI machine to 40 sec (normal is 16 sec). This will increase sample signal.

B. Clean up by DyeEx Cartridge (Qiagen)

1. Vortex cartridge
2. Snap off bottom tab, loosen cap on cartridge, & place in a collection tube
3. Spin at 750xg for 3 min
4. Transfer cartridge to a new collection tube (label tube with sample ID)
5. Apply sample dropwise to gel
6. Spin at 750xg for 3 min (no cap on cartridge)
7. Throw away cartridge (sample in collection tube)
8. Transfer sample to a 1.5 ml eppendorf tube
9. Dry in speed-vac with heat on high
10. Add 10 µl formamide to each tube and spin down.
